# Supplementary material for: EnzML: multi-label prediction of enzyme classes using InterPro signatures
Source: BMC Bioinformatics. 2012 Apr 25;13:61. doi: 10.1186/1471-2105-13-61 (PMC3483700; doi:10.1186/1471-2105-13-61)
Supplement: Addtional file 5 — The Java code to format the data files, evaluate and predict. The file enzml_java_code.tar.gz contains the Java code used to format database data to ARFF and XML formats, to execute cross and train-test (jackknife) evaluations and to record evaluation results to database. More information is included in the readme.txt file and the Javadoc files. The code can be used with a MySQL database. To use a different database software, other JDBC drivers might be required. [file 1471-2105-13-61-S5.gz › java_code/enzml2011/doc/test/mulan/attributesfilter/ArffGeneratorForFilterTests.html]

ArffGeneratorForFilterTests


---


|  |  |  |
| --- | --- | --- |
| **Overview** \| **Package** \| **Class** \| **Use** \| **Tree** \| **Deprecated** \| **Index** \| **Help** | |  |
| PREV CLASS   **NEXT CLASS** | **FRAMES**    **NO FRAMES**     **All Classes** |  |
| SUMMARY: NESTED \| FIELD \| CONSTR \| METHOD | DETAIL: FIELD \| CONSTR \| METHOD |  |


---


## test.mulan.attributesfilter Class ArffGeneratorForFilterTests

```
java.lang.Object
  test.mulan.attributesfilter.ArffGeneratorForFilterTests
```

---

``` public class ArffGeneratorForFilterTests extends java.lang.Object ```

---

**Constructor Summary**

`ArffGeneratorForFilterTests()`


| **Method Summary** | |
| --- | --- |
| `int` | `generateArff(java.lang.String dataProps)`             Generate an arff file and return the arff record id |
| `int` | `generateTestArff()`             Generate the old test arff and return the arff record id |
| `int` | `generateTrainArff()`             Generate the old train arff and return the arff record id |

**Methods inherited from class java.lang.Object**

`equals, getClass, hashCode, notify, notifyAll, toString, wait, wait, wait`

**Constructor Detail**

### ArffGeneratorForFilterTests

```
public ArffGeneratorForFilterTests()
```


**Method Detail**

### generateArff

```
public int generateArff(java.lang.String dataProps)
```

:   Generate an arff file and return the arff record id

    :   **Returns:**

---


### generateTestArff

```
public int generateTestArff()
```

:   Generate the old test arff and return the arff record id

    :   **Returns:**

---


### generateTrainArff

```
public int generateTrainArff()
```

:   Generate the old train arff and return the arff record id

    :   **Returns:**


---


|  |  |  |
| --- | --- | --- |
| **Overview** \| **Package** \| **Class** \| **Use** \| **Tree** \| **Deprecated** \| **Index** \| **Help** | |  |
| PREV CLASS   **NEXT CLASS** | **FRAMES**    **NO FRAMES**     **All Classes** |  |
| SUMMARY: NESTED \| FIELD \| CONSTR \| METHOD | DETAIL: FIELD \| CONSTR \| METHOD |  |


---
